# Number and type of *TET2* mutations in chronic myelomonocytic leukemia and their clinical relevance

**DOI:** 10.1038/bcj.2016.82

**Published:** 2016-09-23

**Authors:** M M Patnaik, M F Zahid, T L Lasho, C Finke, R L Ketterling, N Gangat, K D Robertson, C A Hanson, A Tefferi

**Affiliations:** 1 Division of Hematology, Department of Medicine, Mayo Clinic, Rochester, MN, USA; 2 Department of Laboratory Medicine and Pathology, Mayo Clinic, Rochester, MN, USA; 3 Department of Molecular Pharmacology and Experimental Therapeutics, Mayo Clinic, Rochester, MN, USA

*TET2*, located on chromosome 4q24, is frequently mutated (~60%) in patients with chronic myelomonocytic leukemia (CMML).^1, 2^ *TET2* has 11 exons, and variations, especially in exon 3 have been described as a part of age-related clonal hematopoiesis.^3^ In a large population-based study (*n*=17 182), somatic variations involving *DNMT3A*, *TET2* and *ASXL1* were seen in ~11% of the population >80 years of age, and in comparison with patients without clonal hematopoiesis, were associated with an increased risk of hematological malignancies (HR- 11.1) and all-cause mortality (HR-3.7).^3^ In CMML, thus far, clonal *TET2* mutations in the absence of clonal *ASXL1* mutations (*ASXL1*wt/*TET2*mt) have been associated with favorable outcomes.^1, 4^ The exact mechanism behind this interaction remains to be elucidated, one potential explanation being better responses to hypomethylating agents.^4^ In the current larger CMML patient cohort (*n*=261), we describe the number and type of *TET2* mutations and examine their phenotypic and prognostic effects.

Two hundred and sixty one patients with CMML were included in the study. All patients had bone marrow (BM) biopsies and cytogenetics performed at diagnosis. Targeted capture assays were carried out on BM DNA specimens obtained at diagnosis for the following genes: *TET2, DNMT3A, IDH1, IDH2*, *ASXL1, EZH2, SUZ12*, *SRSF2, SF3B1, ZRSR2, U2AF1*, *PTPN11, Tp53, SH2B3, RUNX1, CBL, NRAS, KRAS, JAK2, CSF3R, FLT3, KIT, CALR, MPL*, *NPM1, CEBPA, IKZF* and *SETBP1,* by previously described methods.^1^ *TET2* (NM_001127208.2) coverage extended from exons 3–11, with frame shift, nonsense and missense variations considered pathogenic. Previously annotated single nucleotide polymorphisms (http//www.hapmap.org) were considered non-pathogenic. The 2008 and 2016 World Health Organization (WHO) criteria were used for CMML diagnosis and classification.^5^

Among the 261 study patients, 65% were males and median age was 70 years (range, 28–91). One hundred and fifty four (59%), 64 (25%) and 43 (16%) patients were classified as CMML-0, 1 and 2, respectively. At a median follow-up of 23 months, 174 (67%) deaths and 37 (14%) leukemic transformations were documented. Mutational frequencies included: *ASXL1* 45%, *TET2* 43%, *SRSF2* 40%, *NRAS* 14%, *SETBP1* 13%, *CBL* 10%, *JAK2* 7%, *RUNX1* 6%, *DNMT3A* 6%, *U2AF1* 6%, *SF3B1 5*%, *ZRSR2* 4%, *Tp53* 4%, *IDH2* 4%, *KRAS* 3%, *c-KIT* 3%, *PTPN11* 3% and <1% each for *FLT3*ITD, *CALR* and *MPL*. There were no *IKZF, STAG2* or *SH2B3* mutations seen.

Two hundred and sixty four *TET2* mutations were seen in 113 (43%) patients; these included 34 (30%) patients with frameshift, 30 (27%) with nonsense and 13 (10%) with missense mutations, whereas 36 (33%) patients had more than one type of mutation (Figure 1). Overall, 58 (52%) patients had more than 1 *TET2* mutation: 55 (49%) patients had 1, 47 (41%) had 2 and 11 (10%) had ⩾3 *TET2* mutations. The median variant allelic fractions (VAF) for *TET2* mutations included; frameshift 43% (range, 10–92%), nonsense 47% (range, 9–100%) and missense 47% (range, 14–95%), respectively.

Among the 113 *TET2* mutated patients, 65% were males, and median age was 71 years with no significant difference in age and gender distribution between mutated and un-mutated cases, or type of *TET2* mutations; however, older patients were more likely to carry multiple *TET2* mutations (*P*=0.01) (Table 1). The frequency distribution of *TET2* mutations with age was: <50 years *n*=15 (6%), 50–59 years *n*=25 (10%), 60–69 years *n*=83 (31%), 70–79 years *n*=104 (39%) and ⩾ 80 years *n*=37 (14%), respectively. The cytosine-to-thymidine (C:G> T:A) base pair change (transition) is often considered a somatic mutational signature of ageing.^6, 7^ In this cohort, C>T base pair changes proportionally comprised, 0% <50 years, 18% 50–60 years, 44% 60–69 years, 41% 70–79 years and 50% ⩾ 80 years. In addition, 73% of patients with *TET2* C>T base pair changes had more than one *TET2* mutation. *DNMT3A* mutations significantly clustered with *TET2* C>T base pair changes (*P*=0.03), with 5 of 6 (83%) *DNMT3A*-mutated patients having concomitant *TET2* C>T base pair changes. Incidentally, only 2 of 6 (33%) *DNMT3A* mutations themselves were as a result of C>T base pair changes.

Compared with their un-mutated counterparts, *TET2*-mutated cases were less likely to have a low hemoglobin (*P*<0.001), include CMML-2 (*P*=0.007), have circulating immature myeloid cells (*P*=0.001), have peripheral blood (*P*=0.009) and BM blasts (*P*=0.009), and have higher-risk stratification per clinical, cytogenetic and molecularly inclusive CMML prognostic models (Table 1); these differences were not affected by the type or number of *TET2* mutations. *TET2* mutated cases were more likely to have a higher frequency of *SRSF2* (*P*=0.004) and a lower frequency of *ASXL1* (*P*=0.03), *Tp53* (*P*=0.04) and *IDH1/2* mutations (*P*<0.001); these associations were also not affected by the type or number of *TET2* mutations.

Median survival for the entire cohort (*n*=261) was 24 months. In univariate analysis, survival was superior in *TET2*-mutated (median 33 months) versus wild-type (median 21 months) patients (*P*=0.03; HR 1.3 95% CI 1.12–1.86). This survival difference remained significant after adjustment for age (*P*=0.04), leukocyte count (*P*=0.017), absolute monocyte count (*P*=0.02), absolute lymphocyte count (*P*=0.02), platelet count (*P*=0.015), circulating immature myeloid cells (*P*=0.03), *DNMT3A* (*P*=0.02) and *ASXL1* (*P*=0.045) mutations; however, significance was lost after adjustment for abnormal karyotype (*P*=0.32) and the Mayo Molecular Model (*P*=0.0003). These observations were not affected by the type or number of *TET2* mutations. Finally, our previous observation regarding the survival advantage of *ASXL1wt/TET2mt* versus other genotypes was most apparent for patients with multiple *TET2* mutations (*P*=0.02).^1^

*TET2* mutations are frequent in CMML (~45%) and constitute approximately equal proportions of frameshift and nonsense mutations, while missense mutations are less frequent. Majority of *TET2*-mutated CMML cases harbor more than one mutant variant. Regardless, the relevance of type and number of *TET2* mutations in CMML was limited to an association between older age and number of mutations, and the latter with possibly improved survival in the absence of clonal *ASXL1* mutations.

## Figures and Tables

**Figure 1:**
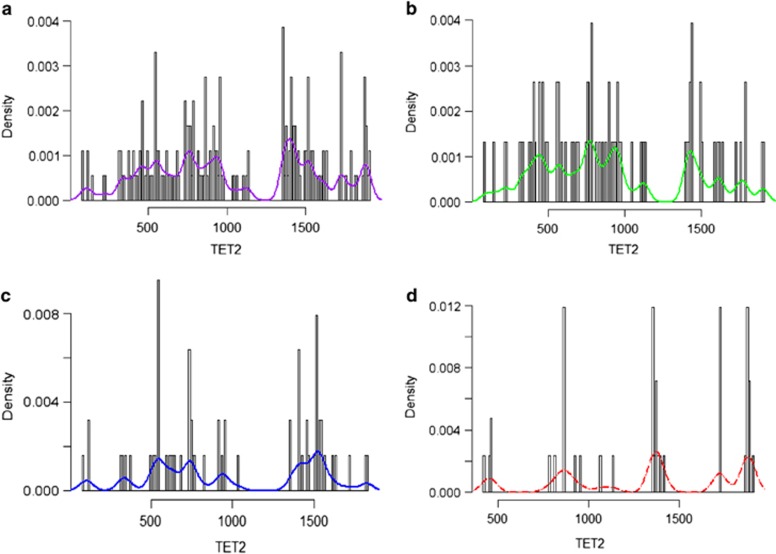
Characterization of *TET2* mutations. Each plot is generated using all mutations from their respective categories. The relative proportion of the mutation subtype is shown on the *y* axis, across the length of the *TET2* gene, from 0 to 2002 amino acids. The colored bar represents the density of the mutations along the gene. (**a**) All mutation types, (**b**) frameshift mutations, (**c**) nonsense mutations and (**d**) missense mutations.

**Table 1:** Clinical and laboratory features and subsequent events in 261 patients with World Health Organization defined chronic myelomonocytic leukemia (CMML), stratified by the presence or absence of *TET2* mutations

*Variable*	*All patients with CMML (*n=*261)*	*CMML patients with* TET2 *mutations (*n=*109)*	*CMML patients without* TET2 *mutations (*n=*152)*	P*-value*
Age in years; median (range)	70 (20–91)	64.5 (20–87)	70 (27–91)	0.067
Males; *n* (%)	168 (64)	9 (56)	159 (65)	0.48
Hemoglobin g/dl; median (range)	10.6 (6.4–16.9)	9.6 (6.8–13.2)	10.7 (6.4–16.9)	0.093
MCV femtoliter; median (range)	91 (59–119)	91 (75–112)	91 (59–119)	0.5
WBC × 10^9^/l; median (range)	12.1 (1.5–264)	12.6 (2.9–71.5)	12 (1.5–264)	0.83
ANC × 10^9^/l; median (range)	5.8 (0–151)	6.7 (1–39.2)	5.7 (0–151)	0.74
AMC × 10^9^/l; median (range)	2.3 (1.0–40)	1.7 (1.0–20)	2.4 (1.0–40)	0.756
ALC × 10^9^/l; median (range)	1.7 (0–22)	1.9 (0.4–5.6)	1.7 (0–22)	0.82
Platelets × 10^9^ /l; median (range)	97 (10–840)	112 (11–840)	96 (10–726)	0.45
Presence of circulating immature myeloid cells; *n* (%)	142 (54)	9 (60)	133 (55)	0.7
PB blast % median (range)	0 (0–19)	0 (0–19)	0 (0–7)	0.3
BM blast % ; median (range)	3 (0–19)	3 (0–13)	3 (0–19)	0.9
BM cellularity %	80 (40–100)			
Lactate dehydrogenase levels IU/ml; *n* (range)	225 (84–1296)	223 (109–294)	225 (84–1296)	0.48
				
*Next-generation sequencing analysis;* n *(%)*				
*** ***Epigenetic regulators				
*** **DNMT3A*	(45)	(50)	(45)	0.7
*** **IDH1*	4 (2)	0 (0)	4 (2)	0.6
*** **IDH2*	11 (4)	0 (0)	11 (4)	0.38
*** ***Chromatin regulation				
*** **ASXL1*	120 (50)	6 (37)	114 (51)	0.3
*** **EZH2*	3 (1)	0 (0)	3 (1)	0.656
*** **SUZ12*	0	0 (0)	0 (0)	−
*** **Transcription factors*				
*** ***RUNX1	16 (6)	2 (12)	14 (6)	0.27
*** ***Spliceosome components				
*** **SF3B1*	13 (5)	4 (25)	9 (4)	**0.0001**
*** **SRSF2*	105 (40)	1 (6)	104 (42)	**0.0042**
*** **U2AF1*	16 (6)	2 (12)	14 (6)	0.2
*** **ZRSR2*	5 (3)	0 (0)	5 (2)	0.8
*** ***Cell signalling				
*** **JAK2 V617F*	17 (7)	1 (6)	16 (7)	0.9
*** **CALR*	1 (0.5)	0 (0)	1 (0.5)	0.8
*** **MPL*	1 (0.4)	0 (0)	1 (0.5)	0.8
*** **SH2B3*	1 (0.5)	0 (0)	1 (0.5)	0.8
*** **CBL*	25 (10)	0 (0)	25 (10)	0.4
*** **KRAS*	8 (3)	0 (0)	8 (3)	0.5
*** **NRAS*	37 (14)	2 (16)	35 (14)	0.8
*** **PTPN11*	6 (2)	2 (12)	4 (2)	**0.005**
*** **CSF3R*	3 (1)	0 (0)	3 (1)	0.7
*** **C-KIT*	7 (3)	1 (6)	6 (2)	0.4
*** **FLT3TKD*	1 (0.5)	0 (0)	1 (0.5)	0.8
*** **NPM1*	0	0 (0)	0 (0)	−
*** ***Tumor suppressor gene*s*				
** ***Tp53*	10 (4)	2 (12)	9 (4)	0.09
** ***PHF6*	0	0 (0)	0 (0)	−
*** ***Others				
** ***SETBP1*	34 (13)	2 (12)	32 (13)	0.9
** ***IKZF*	0	0 (0)	0 (0)	−
				
*2008 WHO morphological subtypes;* n *(%)*				
** **CMML-1	221 (84)	13 (81)	208 (85)	0.7
** **CMML-2	40 (16)	3 (19)	37 (15)	
				
*2016 WHO morphological subtypes; n (%)*				
** **CMML-0	154 (59)	10 (62)	144 (59)	0.9
** **CMML-1	65 (25)	4 (25)	61 (25)	
** **CMML-2	42 (16)	2 (12)	40 (16)	
				
a*Spanish Cytogenetic risk stratification;* n *(%)*				
*** ***Low	180 (72)	11 (69)	169 (72)	0.1
*** ***Intermediate	43 (17)	1 (6)	42 (18)	
*** ***High	27 (11)	4 (25)	23 (10)	
				
a*Mayo-French cytogenetic risk stratification;* n *(%)*				
*** ***Low	180 (72)	11 (69)	169 (72)	0.4
*** ***Intermediate	57 (23)	3 (19)	54 (23)	
*** ***High	13 (5)	2 (12)	11 (5)	
				
*Mayo prognostic model;* n *(%)*				
*** ***Low	89 (34)	3 (20)	86 (35)	0.2
*** ***Intermediate	83 (32)	4 (27)	79 (32)	
*** ***High	87 (34)	8 (53)	79 (32)	
				
*Molecular Mayo model;* n *(%)*				
*** ***Low	26 (10)	0 (0)	26 (11)	0.5
*** ***Intermediate-1	72 (28)	4 (25)	68 (28)	
*** ***Intermediate-2	79 (30)	6 (37)	73 (30)	
*** ***High	81 (31)	6 (37)	75 (31)	
				
*GFM CMML prognostic model;* n *(%)*				
*** ***Low	119 (46)	9 (56)	110 (45)	0.4
*** ***Intermediate	92 (36)	6 (37)	86 (35)	
*** ***High	48 (18)	1 (6)	47 (19)	
Leukemic transformations; n (%)	37 (14)	4 (25)	33 (13)	0.2
*** ***Deaths; *n* (%)	174 (67)	10 (62)	164 (67)	0.7

Abbreviations: ALC, absolute lymphocyte count; AMC, absolute monocyte count; ANC, absolute neutrophil count; BM, bone marrow; CMML, chronic myelomonocytic leukemia; FAB, French American British; GFM, Groupe Français des Myélodysplasies; MCV, mean corpuscular volume; PB, peripheral blood; WBC, white blood cell count; WHO, World Health Organization.

a Cytogenetic studies were available for 250 patients with chronic myelomonocytic leukemia at diagnosis.
